# Breast cancer histopathology, classification and clinical management: Current perspectives

**DOI:** 10.6026/9732063002002069

**Published:** 2024-12-31

**Authors:** Ravindra Waykar, Srinivasakumar Kumarapillai

**Affiliations:** 1 Department of Pharmacy, Lincoln University College, Wisma Lincoln, Jalan SS 6/12, 47301 Petaling Jaya, Selangor Darul Ehsan, Malaysia

**Keywords:** Breast cancer, classification, heterogeneity, molecular mechanisms, ductal carcinoma in situ, invasive ductal carcinoma

## Abstract

Breast cancer (BC) manifests as a diverse group of malignancies and presents as a wide array of tumors with distinct morphological, biological and clinical characteristics. Molecular classification of BC serves as the basis for current precision-oriented therapeutic strategies. Upcoming therapeutic strategies will emphasize personalized medicine and tailoring treatments according to each patient's specific needs. These approaches will involve modulating the therapy intensity based on the biological characteristics of tumours and early predictive indicators, allowing for more precise and adaptable care in oncology. Additionally, there remains an unfulfilled requirement for the creation of new medications to treat breast cancer in its early stages, as well as in advanced cases. This review article presents an extensive examination of breast cancer, delving into its prevalence, contributing factors, molecular and cellular features and therapeutic interventions.

## Background:

In recent decades, the incidence of breast cancer has increased, cementing its position as the most frequently identified cancer type worldwide [1]. Million new cases of this disease were reported in 2008; nearly 60% of these deaths and nearly half of all cases occurred in lower-income nations [2]. Globally, the estimated 5-year survival rate for breast cancer (BC) varies significantly between high- and low-income countries; in the former, it is less than 40%, whereas in the latter, it is 80% [3]. Low- and middle-income nations have limited infrastructure and resources, making it difficult to achieve the objective of enhancing results for breast cancer using early identification, treatment and detection [4]. The lifetime of BC in an American female is 1 out of 8 or 12.5 % [5]. Cancer risk assessment models categorize women's likelihood of developing cancer by evaluating established and measurable risk factors, including hormonal, environmental, personal and genetic elements, which provide tailored screening recommendations based on individual risk profiles [6]. Although magnetic resonance imaging and ultrasound have emerged as valuable diagnostic tools, mammography remains the primary method for breast cancer screening and diagnosis [7]. Unlike other cancers, breast cancer has distinct risk factors and genetic susceptibility plays a key role in its growth [8]. Mutations in BRCA1 or BRCA2 are important benefactors to BC [9]. Breast tumors may originate in several regions of the breast including ducts, lobules and interstitial tissue [10]. Among the extensive array of heterogeneous breast carcinomas, several forms of breast cancer are classified according to their invasiveness in relation to the main tumor locations [11]. This establishes the foundation for further morphological characterization and breast cancer categorization to predict therapeutic outcomes [12]. Therefore, this review provides a thorough analysis of the key biological aspects of BC, including etiological factors, categorizations, features at the cellular and molecular levels and multidisciplinary approaches to BC treatment.

## Breast cancer risk-prediction model - BOADICEA:

Breast cancer risk-prediction models typically categorize women into different risk levels based on various factors. These categories generally include the low, intermediate and high-risk categories [13]. BOADICEA is a predictive algorithm for assessing breast cancer risk [14]. This innovative approach to predict breast cancer risk is a significant step forward by combining various risk factors, including lifestyle, reproductive, hormonal and genetic factors. By utilizing this holistic method, the model enables a more precise and individualized risk evaluation. The risk of developing BC is substantially affected through intrinsic hormones and reproductive-related factors [15, 16]. BC risk in postmenopausal females is significantly influenced by internal hormones, with estrogens and androgens playing crucial roles [17]. Reproductive factors have also been implicated, potentially through their ability to modulate hormone exposure across a woman's lifespan [18]. Recent advancements in risk prediction models have demonstrated the potential benefits of incorporating hormone measurements, leading to improved identification of women at great risk of BC [19].

## Histology of breast carcinoma:

The majority of BC originates in the lobules or ducts of the breast. At times, malignant growth extends into the dermal layer or thoracic wall structures such as the chest muscles. Additionally, cancer cells can modify their surrounding environment, creating conditions that support their proliferation and spread.

## Ductal carcinoma in situ (DCIS):

DCIS is a non-invasive BC marked by the proliferation of atypical epithelial cells within the breast milk ducts, remaining restricted by the basement membrane without invading adjacent tissues [20]. It is fundamentally a precancerous lesion regarded as a precursor to invasive BC, frequently identified via mammograms due to calcification patterns and categorized according to histological characteristics, such as grade (low, intermediate, high) and architectural configuration (solid, cribriform, papillary, micropapillary) [21]. DCIS is classified according to the morphology of cancer cells, encompassing nuclear dimensions, configuration and mitotic frequency, with low-grade cells resembling normal cells more closely, whereas high-grade cells have more aggressive characteristics. The hallmark of DCIS is that the atypical cells remain entirely confined inside the ductal structures without infiltrating the adjacent breast tissue [22]. The basement membrane, which delineates the ductal epithelium from the adjacent tissue, remains preserved in DCIS. Under microscopic examination, DCIS shows substantial proliferation of epithelial cells inside the ducts, frequently appearing congested and disordered in contrast to normal breast tissue [23]. The histological characteristics of DCIS, especially the grade and size of the lesion, profoundly affect the treatment choices. High-grade DCIS exhibiting specific architectural patterns may provide an elevated risk to invasive BC, warranting enhanced surveillance and may be a more aggressive intervention [24].

## Lobular carcinoma in situ (LCIS):

LCIS is a benign condition depicted by the proliferation of atypical cells within the breast lobules, signifying an intensified risk of invasive BC. However, it is not classified as cancer; it typically necessitates vigilant observation and enhanced screening owing to this heightened risk [25]. Following the diagnosis of classic LCIS, the risk of invasive carcinoma is roughly 9-10 instances greater than that in general human beings [26]. This condition is not identifiable through visual examination and is typically found incidentally in breast samples or biopsies conducted for other reasons [27]. Under microscopic examination, LCIS generally maintains its fundamental structure and appears as lobules. The enlarged lobules were filled with a non-cohesive pattern of mid-range cells, characterized by a largely uniform population of round, normochromic nuclei. Intracellular mucin droplets are commonly observed and occasionally accompanied by signet ring nuclei [28].

## Invasive ductal carcinoma (IDC):

IDC is the predominant type of BC, approximately estimated eighty percentages of all BC cases [29]. IDC cancer cells penetrate through the walls of the milk ducts and infiltrate the adjacent breast tissue [30]. IDC frequently occurs alongside DCIS and this combination (IDC + DCIS) correlates with improved overall survival compared to IDC alone. The occurrence of DCIS in patients with IDC correlates with advantageous clinical attributes, including reduced T/N stage, low/intermediate grade and progesterone receptor (PR)/estrogen receptor (ER) positivity. This survival advantage is restricted to individuals with invasive tumors < 4 cm or those with node-negative disease [31]. Furthermore, understanding the molecular distinctions between DCIS and IDC, together with the influence of matrix stiffness on cancer progression, may facilitate the development of more focused treatments and enhance patient outcomes [32, 33].

## Invasive lobular carcinoma (ILC):

ILC is the 2nd major invasive mammary cancer that differs physiologically referred to as the invasive lobular type [34]. ILC tumor cells show a typical development pattern with single-file stroma invasion. They are usually spherical, tiny, no cohesive and rather homogenous. Certain cyto-architectural features can be used to diagnose ILC [35]. The hallmark cyto-architectural characteristics of ILC are expressed by the classic type of ILC. These characteristics typically include the presence of uniform, small tumor cells scattered individually throughout the stroma, creating patterns and lobules surrounding the cells in a circular (targetoid) arrangement [36]. It is typical to observe foci of stromal elastosis surrounding veins and ducts with varying lymphocytic infiltrates. This variation does not have glandular differentiation. ILC seems to be on the rise, especially in postmenopausal women and hormone replacement therapy may be somewhat to blame for this development [37]. ILC is frequently associated with molecular changes that lead to the disappearance of heterozygosity and methylation, with mutations that inactivate E-cadherin, particularly the pleomorphic subtypes [38].

## Uncommon breast malignancies - clinical and pathological features:

Uncommon breast malignancies exhibit diverse clinical and pathological features that distinguish them from more common types such as invasive ductal and lobular carcinomas. Metaplastic breast carcinoma (MBC) is an uncommon BC, distinguished by the conjunction of carcinoma and non-epithelial elements [39].

## Papillary thyroid carcinoma (PTC):

PTC is the utmost known type of thyroid cancer and an endocrine malignancy [40, 41-42]. These vicious distinct include hobnail, tall cell and columnar cell variants, which may lead to metastases, recurrence and death in 10-15% of patients [43]. The different PTC variants exhibited distinct molecular profiles. The follicular variant of PTC shows a higher prevalence of ras mutations (43%) than non-follicular variants (0%), whereas ret/PTC redisposition is well known in non-follicular variants (28% vs. 3%) [44]. BRAF p.V600E mutation is the most common mutation in PTC, including the hobnail variant [45]. Additionally, microRNAs, such as let-7a, play a role in PTC progression by targeting genes such as AKT2 [46]. Recent genomic studies have expanded our understanding of PTC, identifying new driver alterations (EIF1AX, CHEK2 and PPM1D) and distinct gene combinations [47]. These findings have diminished the proportion of PTC occurrences with unspecified oncogenic forms. Furthermore, DNA methylation analysis revealed associations between specific methylation patterns and lymph node metastasis in PTC, with genes such as NDRG4, FOXO3, ZEB2 and CDK6 showing differential methylation [48]. These molecular insights provide a basis for reclassifying thyroid cancers.

## Metaplastic breast carcinoma (MBC):

MBC is an uncommon and aggressive subdivision of invasive mammary carcinoma, distinguished by the distinction of epithelium neoplastic into mesenchymal-looking elements and/or squamous cells [49]. It exhibits poor outcomes and suboptimal responses to systemic chemotherapy compared with standard invasive ductal carcinomas [50]. MBC is typically triple-negative and deficient in the expression of HER2, progesterone receptor (PR) and the estrogen receptor (ER) [51, 52]. MBC often presents with imaging features that are less suggestive of malignancy than invasive ductal carcinoma. MBC masses are more likely to be oval shaped with circumscribed margins rather than irregular with spiculated margins [53]. Sonographically, MBC frequently appears as a dark tissue mass due to its complex echogenicity and posterior enhancement [54]. These benign-appearing features can complicate the diagnosis and may lead to misclassification [55, 56]. The unique histological and molecular characteristics of MBC contribute to its aggressive behavior and poor prognosis. Recent studies have identified potential therapeutic targets such as the PI3K/mTOR pathway and TRIM24 [57, 58-59]. The development of mouse models such as the Ccn6fl/fl; MMTV-Cre model provides valuable tools for studying MBC pathogenesis and testing new treatment strategies [60]. Given the rarity of MBC, further research is needed to optimize diagnosis and treatment approaches for this challenging breast cancer subtype.

## Apocrine carcinoma:

Apocrine carcinoma is a type of BC characterized by distinct histochemical, immunological, morphological and molecular genetic features [61]. It typically presents as a painless, firm, or cystic nodule, with the axilla being the most common site, although it can also occur in various other locations [60]. The determination of apocrine malignancy requires a combination of morphology in over ninety percent of tumor cells and a specific immuno-histo-chemical profile [63]. There are contradictions in the literature regarding the prognosis and origin of apocrine carcinomas. While one study found a slightly longer median survival for apocrine carcinoma patients than for those with nonspecific duct carcinomas, the ultimate outcome was identical [64]. Additionally, the iron reaction test used to identify true apocrine glands was negative in all cases, suggesting that resemblance to apocrine glands may be purely morphological. However, recent molecular classifications have identified subsets of breast tumors with high androgen receptor expression, including "luminal androgen receptor (LAR) tumors" and "molecular apocrine tumors" (MATs), which may have implications for targeted therapies [63]. Apocrine carcinoma remains a challenging diagnosis because of the subjectivity of histopathological criteria and lack of specific biomarkers [65]. Treatment options include wide local excision with consideration for lymph node dissection in cases of confirmed metastases or aggressive tumors [66]. Although traditionally resistant to chemotherapy and radiation, recent research suggests that drug treatments for breast cancer, including anti-HER2 and hormone therapies, may be effective for some apocrine carcinomas [67]. Further studies are required to improve our understanding of this rare cancer and develop standardized treatment protocols [68].

## Adenoid cystic carcinoma (ACC):

ACC is a tumor that affects the salivary glands and may be found at sites such as the lacrimal glands, upper respiratory tract and skin [69, 70]. It is characterized by slow growth and distant metastasis, often leading to poor long-term prognosis [71]. ACC can arise in unusual locations, including the larynx, prostate and external auditory canal, making diagnosis challenging [72]. ACC exhibits diverse clinical behaviors, depending on its location. Although salivary gland ACCs are generally aggressive, cutaneous ACCs may have a more indolent course [73]. However, the histological and immune-cytochemical features of ACCs from different sites appear identical, suggesting a uniform pathological entity [74]. Another intriguing aspect is the potential for dedifferentiation in ACC, which is associated with an accelerated clinical course and may involve modifications in the p53 gene. ACC remains a poorly understood malignancy, with limited treatment options. Standard therapy includes surgery and radiation, but the propensity for distant metastases limits survival [75]. Novel approaches, such as targeted therapies like anlotinib, show promise in advanced cases [76]. Additionally, high PSMA expressions in ACC tumors suggest that 68Ga-PSMA PET-CT could be a valuable imaging tool for this malignancy [77]. Further research on the molecular mechanisms of driving ACC is crucial for developing more effective treatments and improving patient outcomes.

## Breast carcinomas with endocrine differentiation:

These tumors are typically hormone receptor-positive and express ER and/or PR, making them candidates for endocrine therapy [78, 79]. However, some endocrine-differentiated breast cancers may lack ER and PR expression while still expressing the androgen receptor (AR), as observed in apocrine carcinomas [80, 81]. Interestingly, apocrine carcinomas, which are ER-/PR-/AR+ invasive ductal carcinomas, often show different immune-histo-chemical profiles than other breast cancer subtypes. For instance, although TRPS1 is typically a sensitive marker for invasive breast carcinoma, it is frequently negative in apocrine carcinomas. In contrast, GATA3 remains positive in these tumors, regardless of HER2 status [82]. This distinction is critical for diagnosis and classification of BC with endocrine differentiation. Breast carcinomas with endocrine differentiation encompass a spectrum of tumors with varying hormone receptor profiles. Although most are hormone receptor-positive and responsive to endocrine therapy, some subtypes, such as apocrine carcinomas, may require different treatment approaches. Understanding the molecular and immune-histochemical characteristics of these tumors is crucial for their proper diagnosis, classification and treatment selection [83, 84].

## Phyllodes tumors (PT):

PT is biphasic tumors consisting of epithelial and stromal components, with the ability to recur and metastasize [85]. Interestingly, although PTs are typically benign, both stromal and epithelial components can progress to malignancy [86]. In rare cases, carcinoma may develop within a PT with the potential for lymph node metastasis [87]. The differential diagnosis between PT and fibroadenoma remains challenging as it exhibits a continuum of pathological features [88]. Molecular studies have revealed that genetic changes are the most consistent finding in comparative genomic hybridization [89]. The accurate diagnosis and classification of PTs are crucial for appropriate clinical management. While histological assessment remains the primary method for diagnosis, molecular studies and immune-histochemical markers may provide additional insights into tumor behavior and potential therapeutic targets [90].

## Primary breast lymphoma (PBL):

PBL predominantly affects older women [91]. PBL subdivisions, such as "anaplastic large-cell lymphoma (ALCL)", have also been reported, particularly in association with breast implants [92] although B-cell lymphomas are more common overall, T-cell lymphomas have been frequently reported in cases associated with breast prostheses [93]. Additionally, a subgroup of bilateral breast lymphomas has been identified in young women, particularly during pregnancy or the postpartum period [94]. The mutational profile of PBL involves genes in the NF-κB signaling pathway, with PIM1 mutations being notably frequent [95]. The diagnosis of PBL can be challenging owing to its nonspecific imaging features, which often overlap with those of primary breast carcinoma [96]. Treatment typically involves a combination of chemotherapy, immunotherapy and radiotherapy with surgery playing a less significant role than breast cancer management [97]. The prognosis for PBL is generally favorable, with a five-year likelihood of survival of about 76% in non-Hodgkin lymphoma cases [98].

## Breast sarcoma:

Rare and diverse primary breast sarcomas are about one percent of all BC [99]. Most studies on this uncommon malignancy are retrospective case studies and individual accounts, making clinic-pathological analysis difficult [100]. Complete excision with clear margins is recommended for tumors that are < 5 cm in diameter. Preoperative chemotherapy may enhance the margins of bigger tumors [101]. Tumors > 5 cm or those with positive surgical margins require radiation [102]. Despite its modest risk, breast cancer radiation causes angiosarcoma [103]. Two-thirds of breast sarcoma patients die [104]. Smaller tumors at presentation increase survival [105]. Patients with lymphangiosarcoma and other sarcomas have a thirty percent likelihood of survival after treatment [106].

## Breast cancer staging:

The "American Joint Committee on Cancer (AJCC) 8th edition" includes two distinct staging tables for BC: one established on anatomical basis and the other on prognostic factors [107, 108]. The TNM classification system, which delineates the anatomic spread of cancer, serves as the foundation for establishing the anatomic stage. Anatomical staging encompasses the assessment of three crucial elements: the "dimensions of the primary tumor (T), condition of the lymph nodes (N) and existence of distant metastases (M)" [109]. This evaluation was performed using both clinical and pathological methods. The National Comprehensive Cancer Network (NCCN) recommends a series of steps to determine anatomic stage [109]. These include conducting a thorough "history and physical examination, performing bilateral mammography with ultrasound" when indicated, analysing pathology results and evaluating hormone receptor status [109]. The 8th edition of the AJCC staging system encompasses four distinct categories within the anatomic TNM classification. Among these, the first category is known as clinical staging, which is indicated by the prefix "c". This classification relies on the information gathered through clinical examinations, diagnostic imaging procedures and samples collected via core biopsy or aspiration techniques before any treatment is administered. Pathologic staging, indicated by the prefix "p", represents the second category and is derived from the analysis of surgical specimens, which encompasses those obtained through sentinel lymph node biopsy (SLNB). The prefix "yp" denotes the third classification, post-therapy staging, which is applicable to individuals who have undergone neoadjuvant treatment including chemotherapy (NAC), radiation, or hormonal therapy. The final classification, restaging, was employed when a tumor reappeared. Quantitative classification is the foundation of anatomic staging systems. This system categorizes primary tumors from Tis to T4, assesses the regional lymph node status from N0 to N3 and identifies distant metastases as either M0 or M1. By integrating these individual classifications, the overall anatomic stage was determined, spanning from stage 0 to stage IV [110].

## Genetic predisposition in BC:

Almost 10% of BC patients have genetic vulnerability associated with germline mutations. BRCA1/2 mutations play a significant part in the genetic vulnerability of BC. Seventy percent of individuals with BRCA1/2 mutations have a high chance of developing BC at 80 years of age [111]. Several mutations in BRCA1 can cause splicing mistakes during put-up-transcriptional mRNA amendment, culminating in exon 11 deletions. The companion and localizer of BRCA2, PALB2, is frequently reflected as an excessive BC-susceptible gene in conjunction with BRCA1/2. PALB2 is currently recognized as being essential in BC prognostic landscapes and has obtained a decent function in BC predisposition panel tests. TP53 is normally modified in cancer. Nearly 30% of breast tumors contain a TP53 mutation, which varies in frequency and spectrum according to the subtype and race element [112]. The ATM gene, which is not repressed, is crucial for genomic balance [113]. It is activated via double-stranded DNA breaks during the duration of the DNA harm reaction (DDR) [114]. Mutations in ATM are responsible for a rare autosomal recessive disorder known as ataxia-telangiectasia (A-T) [115]. It is exemplified by immunodeficiency, susceptibility to ionizing radiation, neuro-degeneration and an increased likelihood of BC. Individuals with BC who received radiation treatment and possessed mutated ATM experienced secondary malignancies earlier than those who did not undergo radiation therapy and did not have mutated ATM [116, 117]. CHEK2 encodes protein checkpoint kinase 2, which is a regulator of DNA repair that maintains genomic stability. The likelihood of BC is doubled or tripled by protein-truncating mutant CHEK2 [118]. An STK11 mutation reduces the capacity of tumor cells to spark off AMP kinase, resulting in a higher power strain [119]. Moreover, STK11 has an unfavorable impact on the mTOR cascade, which may result in aberrant mTOR signaling. PJS intestinal polyps may also display increased mTOR signaling [120]. The lack of heterozygosity in STK11 causes breast cancer, metastasis and poor diagnosis [121].

## Consequences of epigenetics:

**Figure 1:**
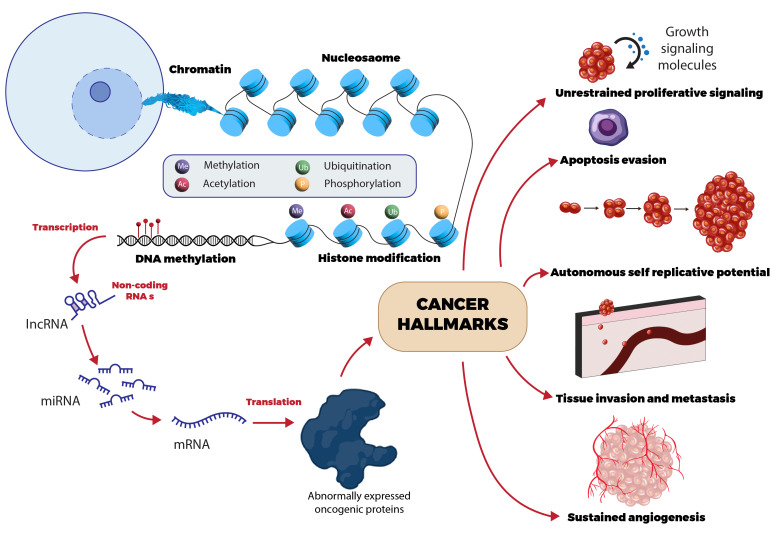
Epigenetics of breast cancer

Genetic and epigenetic abnormalities in BC progression sooner or later contribute to the formation of neoplastic cells (Figure 1) [122]. In oncology, there are multifactorial regulatory mechanisms that force primary tumorigenesis, invasion, or even the application of immune reactions in the tumor environment [123, 124-125].

## DNA methylation:

This is an intrinsic technique caused by an enzyme that attaches CH3 group to either cytosine or adenine [126]. TNBC, the most severe form of breast cancer, exhibits heightened aggressiveness compared to other subtypes [127]. The development of TNBC tumors is thought to be influenced by abnormal epigenetic mechanisms, particularly DNA hyper methylation [128]. This process is facilitated by the enzyme DNA methyltransferase 1 (DNMT1), contributes significantly to the onset and progression of TNBC [129]. Subsequently, it regulates the techniques of genome imprinting, post-translation, transcription and silencing repetitive DNA regions. ER+ cancers are a long way more likely to have altered DNA methylation than ER + tumors [130]. In the evaluation of ER tumors, ER+ tumors were substantially more likely to undergo DNA methylation alterations.

## Histone modification (HM):

Histone proteins often change post-translationally and regulate the chromatin. HM patterns in BC cells are distinct, based on their unique phenotypic traits. The HM enzyme EZH2 is associated with more severe forms of BC [131]. Recent studies have shown that HER2-amplified breast cancer shows enhanced H3 and H4 lysine acetylation [132, 133]. Significant data suggest that luminal BC had higher levels of these HM markers, which improved the prognosis [134]. However, low marker levels predicted poor outcomes in HER2+ and triple-negative breast cancer [135].

## Noncoding RNAs (ncRNAs):

Transcriptomics rapidly identifies disease-related ncRNA functions. These transcripts are categorized into lncRNAs (long ncRNAs) and sncRNAs (small ncRNAs) based on their regulatory features and length, both of which influence gene expression [136]. It exerts a substantial influence on BC development by modulating diverse cellular functions [137]. The rise in ncRNAs affects gene expression and contributes to breast cancer development and lncRNAs, which exceed 200 nucleotides in length, play a role in regulating human gene expression and various physiological and pathological processes. Secondary and tertiary structures may help to attract targets. In breast cancer, lncRNA GAS5 is downregulated. HOX transcript antisense intergenic RNA (HOTAIR) upregulation leads to BC metastasis [138]. Upregulation of metastasis-associated lung adenocarcinoma transcript 1 (MALAT1) is associated with reduced 5-year survival in BC patients. Decreased MALAT1 levels diminished breast cancer invasion and progression [139]. Other regulatory sncRNAs support crucial biological processes through RNA-protein complexes and contribute to cancer development in multiple ways [140]. MiRNAs significantly influence BC pathogenesis. Owing to their cell and tissue specificity, miRNA expression patterns can differentiate between normal and breast cancer samples based on molecular subtype and hormonal status [141]. These multi-marker miRNAs play vital roles in breast cancer prognosis, targeted treatment and efficacy [142, 143-144].

## Signalling pathways in BC:

Hormones usually regulate the proliferation of mammary cells. Cells communicate through diverse signalling pathways [145]. Aberrations in signalling pathways can cause the development and spread [146]. Genetic and epigenetic adjustments impact the tumor microenvironment. Discrepancies in any of these pathways will lead to unpredictable results in other pathways [147]. The following sections highlight the essential signalling pathways and their interactions that govern mammary gland development and breast cancer. A complex stroma encases a densely branched web of epithelial tubes that comprises the mammary glands. An epidermal placode gives rise to mammary epithelium during embryonic development. Ten to twelve primitive ductal components situated underneath the areola-nipple complex constitute the breast rudiment at birth. The presence of mammary stem cells (MaSCs) in situ and unipotent cells that regulate ductal tree homeostasis and morphogenesis has been brought to light by lineage tracing. Additionally, it has been determined that both normal human and mouse mammary tissues include a variety of luminal progenitor subtypes. MaSCs make up a relatively tiny percentage of the undifferentiated cells of the mammary gland, which may divide symmetrically and asymmetrically to generate a range of differentiated cells and create new MaSCs through self-renewal. Breast Cancer stem cell (BCSC) theory proposes a division regarding the nature of cancer stem cells (CSCs): they are either the initial cells from which cancer develops, or they represent malignant cells that have acquired stem cell characteristics. The first perspective is rooted in the observed parallels between tissue regeneration and tumor formation processes, while the alternative suggests the transformation of cancerous cells into stem-like entities.

## Estrogen (ER) signalling pathways:

ER is conventional steroid receptors. Two unique genes, ESR1 and ESR2, encode the alpha (α) and beta (β) isoforms of ER. These receptor elements are transcriptional and induce a series of actions. Although BC cell survival and proliferation are associated with ERβ expression, its specific features are yet to be understood. In response to contact with estrogen, the ER receptor protein fiberizes and accounts for nucleus dislocation, which controls transcriptional activity. In the final step, ER coactivators (CoA) are engaged, which attach in a coordinated manner to the estrogen response element (ERE) sequences within the DNA, initiating the transcription of numerous genes that control signal transduction and cell viability [148]. Although ERα expression is frequently accelerated in breast cancer, its association with ERβ improves the analysis [149]. Postmenopausal women with a relative decrease in estrogen levels undergo metabolic alterations associated with the law of electricity metabolism using ER signaling [150]. A growing number of studies indicate that overexpression of Erα causes 70% of breast cancers, but this is a small percentage. In normal breast tissues, there is an inverse relationship between Erα expression and cellular proliferation, which can be explained by the fact that Erα expression is downregulated when cells enter the mobile cycle. In contrast, Erα cells in benign breast tumors transform precancerous hyperplasia into invasive malignancy via apoptosis, mobile cycle arrest and senescence [151].

## Signaling path for HER2:

**Figure 2:**
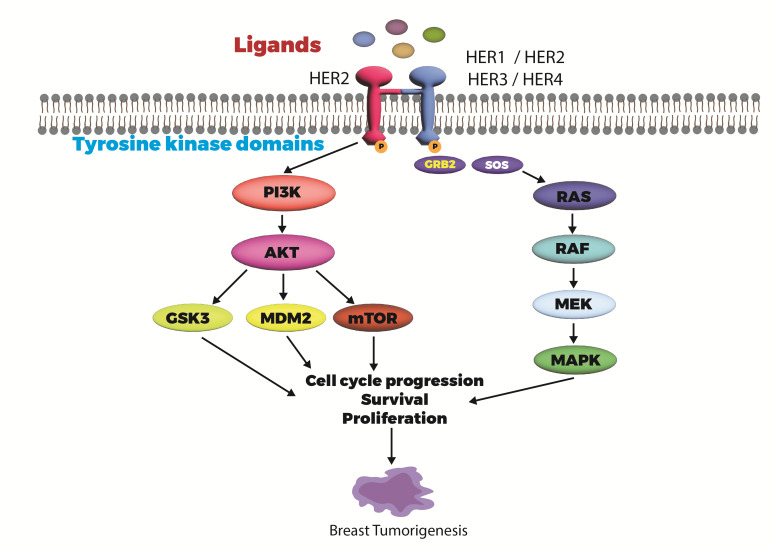
HER pathway

Type I trans-membrane receptors, called human epidermal growth factor receptors (HERs), promote intracellular signaling in response to inputs from the outdoor environment. A ligand can induce homo- or hetero-dimerisation when it binds to HER proteins. Tyrosine residues in the intracellular area are phosphorylated at some point during protein dimerization [152]. Adaptor proteins that might be attracted to phosphorylated residues trigger messenger pathways downstream, including the PI3K/Akt and MAPK pathways (Figure 2) [153, 154]. Furthermore, Akt/mTORC1-mediated HIF-α stimulates VEGF secretion and enhancing angiogenesis [155]. The number common cause of breast cancer is HER2 amplification. Chemotherapeutic drugs are more effective against HER2-tremendous breast cancer cells, which might also be more susceptible to brain metastasis [156]. HER2 overexpression in breast cancers is a prime reason for most cancers, making it treatable [157]. As receptor tyrosine kinases, HER and its fellow EGFR family members are situated on the cellular membrane, where they react with a broad spectrum of ligands. Activation of downstream oncogenic signaling cascades, such as the PI3K/AKT and Ras/MAPK pathways, is triggered by the phosphorylation of the tyrosine kinase domain located in the cytoplasm.

HER/EGFR - Human epidermal growth factor receptors, PI3K - phosphatidylinositol 4,5-bisphosphate 3-kinase, AKT alternatively referred to as protein kinase B (PKB), GSK3 - Glycogen synthase kinase 3, MDM2 - murine double minute, mTOR - mammalian target of rapamycin, GRB2 - Growth factor receptor-bound protein 2, SOS - Son of Sevenless, RAS - Ras protein, RAF - Rapidly Accelerated Fibrosarcoma, MEK/MAPK - mitogen activated protein kinase.

## Notch signaling:

The signaling cascade is important for embryonic improvement and it is possible that both organogenesis and cancer share similar molecular procedures [158]. Notch signaling was first found to be related to BC in MMTV-induced tumors. In vitro atmospheric culture revealed bizarre Notch activation and accelerated NICD and HES1 accumulation, imparting insight into DCIS's molecular characteristics of DCIS. Notch3 stimulates tumor cell self-renewal and aggressive metastasis [159]. The Notch signaling pathway is activated when Del or Jag protein ligands interact with Notch receptors. This interaction triggers proteolytic cleavage and NCID binding, ultimately leading to the transcription of genes involved in angiogenesis.

## AKT/mTOR/PI3K pathway:

The intracellular vesicular trafficking enzyme PI3K is an important signal modulator. Numerous extracellular signals cause PI3K to turn out to be autophosphorylated, which then activates PDK1 and AKT. Mutant PIK3CA is present in twenty to thirty percent of BC patients and in clinical settings, these results in resistance to anti-HER2 medication [160]. Since AKT can nevertheless trigger the ER pathway in the absence of estrogen, similar research has determined that the PI3K/AKT is proof against endocrine remedies. Therefore, resistance may be prevented by combining endocrine therapy with AKT and mTOR inhibitors [161].

## Hedgehog signalling pathway:

Tumor improvement and metastasis are pushed via downstream targets of the Hh pathway, while GLI protein upregulates pro-angiogenic secreted molecules, such as cysteine-rich molecules. GLI protein upregulates seasoned-angiogenic secreted molecules along with neuropilin 2 (NRP2), cysteine-rich angiogenic inducer 61 (CYR61) and VEGFR1 and VEGFR2 co-receptors, even as SHH increases the launched factors [162]. In BC cells, the hVEGF-A gene promoter was upregulated using a shorter GLI1 [163]. In transgenic mouse embryos, excessive Hh signaling can result in aberrant mammary buds [164]. Immunohistochemistry studies have shown that invasive tissues containing carcinomas have accelerated expression levels. However, consistent with the latest studies, BC metastasis was due to the activation of GLI.

## Breast cancer treatments:

Treatment options for BC include chemotherapy, radiation therapy, surgery, targeted therapy and hormonal therapy with the choice depending on factors such as tumor stage, biomarkers and individual patient characteristics [165, 166-167]. Radiation therapy exhibits a crucial part in improving survival, particularly after surgery and in high-risk subjects after mastectomy [168]. Targeted therapies such as HER2-targeted treatments have enhanced the benefits for patients with HER2-positive breast cancer [169]. Hormonal therapy as well as aromatase inhibitors and selective estrogen receptor modulators (SERMs) is effective for hormone receptor-positive breast cancers [170, 171]. Chemotherapy remains a crucial component of treatment, especially for TNBC [172, 173]. The effectiveness of these treatments can vary based on patient age, with older women less likely to receive guideline-concordant care for various treatment modalities [174]. Additionally, emerging research has focused on amino acid metabolism as a potential therapeutic target [175] and on the use of predictive biomarkers to guide personalized treatment decisions [176]. A combination of multiple treatment modalities is often recommended to achieve outcomes in breast cancer management [177, 178, 179-180].

## Conclusion:

This review aims to provide a comprehensive and up-to-date overview of breast cancer, focusing on its current epidemiological trends, identified risk factors, classification systems, prognostic biomarkers and existing treatment options. The substantial rise in both breast cancer incidence and fatality rates over recent decades underscores the critical need for implementing the most effective preventive measures. Advancements in breast cancer patient care and outcomes have been significantly influenced by persistent exploration of prognostic biomarkers and potential targets for biological therapies.

## Authors' contribution:

R.W. and S.K.P. contributed to the conceptualization of the study and design. R.W. carried out the formal analysis and was involved in the data curation and writing the original draft preparation.

S.K.P. took part in writing the review, editing, supervision and project administration. All authors have read and agreed to the published version of the manuscript.

## Data availability:

The data and supportive information are available in the article.
